# 2183. Porin Mutations Potentiate the *in Vitro* Synergy of Meropenem-Vaborbactam (MVB), but not Ceftazidime-Avibactam (CZA) in Combination with Other Antibiotics against KPC-producing *Klebsiella Pneumoniae* (KPC-*Kp*)

**DOI:** 10.1093/ofid/ofad500.1805

**Published:** 2023-11-27

**Authors:** Tara M Rogers, Ellen G Kline, Kevin M Squires, Chelsea Jones, Ryan K Shields

**Affiliations:** University of Pittsburgh, Pittsburgh, Pennsylvania; University of Pittsburgh, Pittsburgh, Pennsylvania; University of Pittsburgh, Pittsburgh, Pennsylvania; UPMC, Pittsburgh, Pennsylvania; University of Pittsburgh, Pittsburgh, Pennsylvania

## Abstract

**Background:**

CZA and MVB are often used in combination with other antibiotics in clinical practice. Our objective was to assess their *in vitro* synergy stratified by predominant *ompK36* genotype: wild-type (WT), or mutants that included an insertion sequence *5* (IS5) or GD duplication (GD).

**Methods:**

15 KPC-*Kp* clinical isolates were selected. Minimum inhibitory concentrations (MICs) were determined by broth microdilution in triplicate. Time-kill analyses were used to test CZA and MVB alone (1 and 4x MIC) and in combination with colistin (COL; 2 mg/L), fosfomycin (FOS; 100 mg/L + 25 mg/L G6P), gentamicin (GEN; 2 µg/mL), meropenem (MEM; 8 mg/L), and tigecycline (TGC; 2 mg/L) against 1x10^8^ cFu/mL. 24 hour log-kill was calculated as log cFu/mL decrease from time 0.

**Results:**

5 isolates each had *ompK36* WT, IS5, or GD. 27% and 73% were KPC-3 and KPC-2, respectively. 100% were susceptible to CZA and MVB. None were resistant to COL. 3 were non-susceptible to GEN, which were all IS5 (**Table 1**).

In combination with CZA 1x MIC, each of COL, GEN, and MEM were bactericidal against all isolates; mean ± standard error (SE) log kills were -7.39 ± 0.3, -6.58 ± 0.93, and -5.81 ± 0.21, respectively (**Figure 1**). Mean log-kills did not vary for CZA + COL, GEN, or TGC against *ompK36* mutants vs WT; however, log-kills were significantly lower for CZA + FOS (-3.78 vs -6.6; p < 0.05) or MEM (-5.4 vs -6.62; p < 0.01) against *ompK36* mutants compared to WT. Synergy rates were reduced from 100% against WT to 80%, 60%, and 40% against mutant isolates for CZA + COL, MEM, and FOS, respectively (**Figure 2**).

MVB 1x MIC in combination with COL, FOS, GEN, and TGC were bactericidal 67, 27, 80, and 47% of the time, with corresponding log kills of -3.96 ± 1.1, -1.56 ± 0.83, -5.05 ± 0.82, and -2.53 ± 0.28, respectively (**Figure 1**). Mean ± SE log kills were significantly greater against porin mutants compared to WT for MVB + COL (-5.57 vs -0.73; p = 0.03), FOS (-3.11 vs 1.54; p = 0.03), or TGC (-2.93 vs -1.73; p = 0.04). Rates of synergy were also higher against porin mutants vs WT for MVB + FOS or COL (**Figure 2)**.

Table 1

**Figure:**
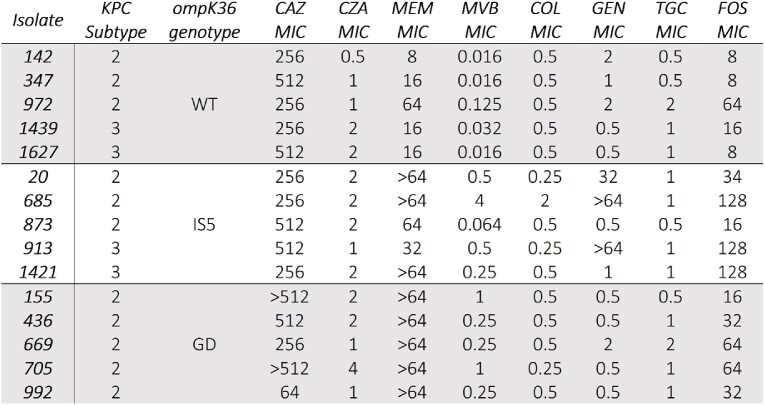

Characteristics and susceptibility data for fifteen KPC-Kp isolates chosen for analysis. CAZ = ceftazidime; COL = colistin; CZA = Ceftazidime-avibactam; FOS = fosfomycin; GD = GD duplication; GEN = gentamicin; IS5 = insertion sequence 5; KPC = Klebsiella pneumoniae carbapenemase; MEM = meropenem; MVB = meropenem-vaborbactam; TGC = tigecycline; WT = wild-type.

Figure 1

**Figure:**
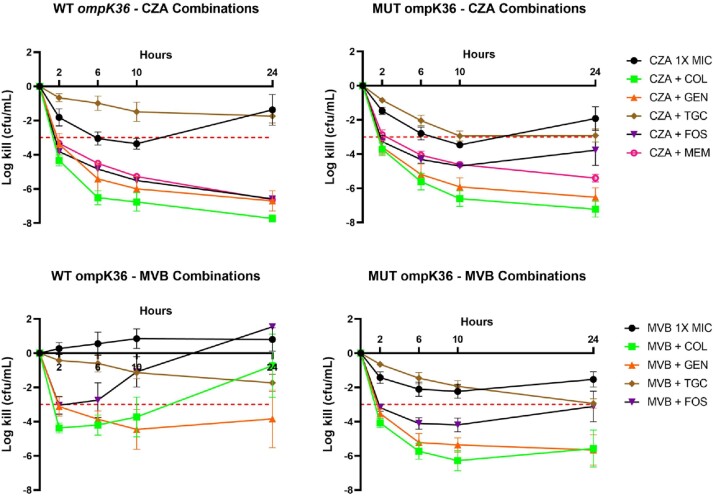

24-hour log kills for antibiotic combinations with CZA (ceftazidime-avibactam) and MVB (meropenem-vaborbactam) at 1X MIC, stratified by ompk36 status: wild-type (WT) versus mutant (MUT). Bactericidal is defined as -3 log kill from time 0 at 24 hours and indicated by the dotted horizontal line. COL = colistin; FOS = fosfomycin; GEN = gentamicin; MEM = meropenem; MIC = minimum inhibitory concentration; TGC = tigecycline.

Figure 2

**Figure:**
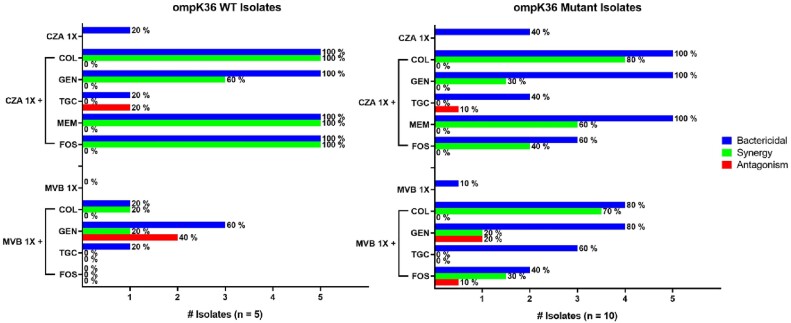

Proportion of bactericidal, synergistic, and antagonistic outcomes for ompK36 wild-type (WT; n = 5) versus mutant (MUT; n = 10) KPC-Kp isolates tested against CZA (ceftazidime-avibactam) or MVB (meropenem-vaborbactam) at 1X MIC as single agents and in combination with colistin (COL), gentamicin (GEN), tigecycline (TGC), meropenem (MEM), or fosfomycin (FOS). Bactericidal activity was defined as -3 log kill from time 0 at 24 hours; synergy and antagonism were defined as -2 or +2 log kill at 24 hours compared to most active single agent, respectively.

**Conclusion:**

Porin mutations negatively impact CZA synergy with other antibiotics compared to WT, though 24-hour log kills for COL, FOS, GEN, and MEM remained below the bactericidal threshold. Conversely, porin mutations potentiate synergy between MVB and COL, FOS, or TGC.

**Disclosures:**

**Ryan K. Shields, PharmD, MS**, Allergan: Advisor/Consultant|Cidara: Advisor/Consultant|Entasis: Advisor/Consultant|GSK: Advisor/Consultant|Melinta: Advisor/Consultant|Melinta: Grant/Research Support|Menarini: Advisor/Consultant|Merck: Advisor/Consultant|Merck: Grant/Research Support|Pfizer: Advisor/Consultant|Roche: Grant/Research Support|Shionogi: Advisor/Consultant|Shionogi: Grant/Research Support|Utility: Advisor/Consultant|Venatorx: Advisor/Consultant|Venatorx: Grant/Research Support

